# Behavior of Healthcare Workers After Injuries From Sharp Instruments

**DOI:** 10.5812/traumamon.12779

**Published:** 2013-08-14

**Authors:** Mohsen Adib-Hajbaghery, Mohammad Sajjad Lotfi

**Affiliations:** 1 Department of Medical-Surgical Nursing, Kashan University of Medical Sciences, Kashan, IR Iran

**Keywords:** Needle stick Injuries, Behaviors, Knowledge, Health Personnel

## Abstract

**Background:**

Injuries with sharps are common occupational hazards for healthcare workers. Such injuries predispose the staff to dangerous infections such as hepatitis B, C and HIV.

**Objectives:**

The present study was conducted to investigate the behaviors of healthcare workers in Kashan healthcare centers after needle sticks and injuries with sharps in 2012.

**Materials and Methods:**

A cross-sectional study was conducted on 298 healthcare workers of medical centers governed by Kashan University of Medical Sciences. A questionnaire was used in this study. The first part included questions about demographic characteristics. The second part of the questionnaire consisted of 16 items related to the sharp instrument injuries. For data analysis, descriptive and analytical statistics (chi-square, ANOVA and Pearson correlation coefficient) SPSS version 16.0 software was used.

**Results:**

From a total of 298 healthcare workers, 114 (38.3%) had a history of injury from needles and sharp instruments in the last six months. Most needle stick and sharp instrument injuries had occurred among the operating room nurses and midwifes; 32.5% of injuries from sharp instruments occurred in the morning shift. Needles were responsible for 46.5% of injuries. The most common actions taken after needle stick injuries were compression (27.2%) and washing the area with soap and water (15.8%). Only 44.6% of the injured personnel pursued follow-up measures after a needle stick or sharp instrument injury.

**Conclusions:**

More than a half of the healthcare workers with needle stick or sharp instrument injury had refused follow-up for various reasons. The authorities should implement education programs along with protocols to be implemented after needle stick injuries or sharps.

## 1. Background

Needle stick injury has been defined as a percutaneous piercing wound by a needle point or other sharp instrument contaminated with blood or body fluids (1). Needle stick injuries are of the most common occupational hazards healthcare workers are faced with (2, 3). These injuries usually occur during activities such as transfusion, blood sampling, needle disposal, waste collection, transferring body fluids and transferring blood from a syringe into another vessel (3). Injuries due to contact with contaminated needles may have serious physical and psychological consequences (4-6). These injuries may be dangerous and predispose healthcare workers to more than 20 different types of pathogens (7) including HIV and hepatitis B and C viruses (8-10). The risk of catching HIV through needle stick is 0.3%; while, such risk is 3% for hepatitis C, and 30% for hepatitis B (8). The risk of exposure is also increases with the increasing number of patients (11). According to the World Health Organization, 16,000 cases of hepatitis C, 66,000 cases of hepatitis B and 1,000 cases of AIDS have occurred through occupational exposure in the year 2000 (12). The costs of injuries related to sharp contaminated instruments in the US has been estimated to be around 118 to 591 million dollars in 2010 (4, 7-13). These injuries also induce considerable psychological aftermaths such as phobia, anxiety and stress in affected individuals (14, 15). It is difficult to provide accurate statistics on the incidence of needle stick or sharps injuries because even in developed countries all cases are not reported (16, 17). Several factors such as time constraint, underestimating the risk, and lack of knowledge may prevent injuries from being reported (18). The incidence of needle sticks among health-care workers varies in different countries. For instance, its prevalence has been reported to be about 66% in Egypt, 45% in Pakistan, 31.4 % in Germany, 46.8% in Saudi Arabia, 45% in Turkey, 50% in Australia and Taiwan and 79.5% in India (19-26). It seems that these injuries are more prevalent in developing countries. In a recent study, 57% of African nurses and midwives had experienced at least one needle stick injury in the past year. Only 18% had not experienced any such injury in their entire career and the rate of needle stick injury was 4.2 per person per year (27); however, the rates of such injuries are lower in healthcare workers of some developed countries (19, 20, 22, 24, 26). The most common causes of needle sticks in various studies were high workload, working hastily, fatigue and a crowded work environment. Also, the highest rates of needle sticks occur during activities such as blood sampling, injections, IV catheter insertion, disposal of contaminated needles, needle recapping and washing contaminated instruments (28, 29). Behaviors and actions of employees after a needle stick are of great importance in preventing the consequences. Healthcare workers should take appropriate actions after needle stick injuries. Also employers are responsible for implementing protocols to reduce such injuries (30). Despite the publication of the guidelines for prevention of sharp percutaneous injuries, such injuries continue to occur. Studies have also shown that compliance with these precautions is less than optimal worldwide (31). Baghcheghi et al., have studied the prevalence of needle sticks and sharps injuries among nursing students and reported that the most important measures taken after needle sticks were washing the area with soap and sending a blood sample to the lab; but in 10% of cases no specific action was taken (28). In another study, Rakhshani et al. studied the prevalence of needle stick injuries among the healthcare professionals in Zahedan hospitals and reported that compression, washing the injured area with povidone iodide and washing with soap were the most prevalent actions respectively. However, in 0.09% of cases nothing was done (29).

## 2. Objectives

Due to the lack of precise information on the rate of needle sticks and sharps injuries and related factors and the differences among previous studies, the present study was conducted to investigate the behaviors of healthcare workers in Kashan’s healthcare centers after needle sticks and sharps injuries.

## 3. Materials and Methods

This cross-sectional study was conducted in the last three months of 2012 on healthcare workers of medical centers affiliated with Kashan University of Medical Sciences. Sample size was calculated based on a previous report of the prevalence of needle sticks and sharps injuries which was 24.1% (32); Three hundred and sixty samples were selected considering a possible attrition rate of 25%. Stratified random sampling was performed. First, the number of staff at each center was assessed. Then, the quota for each center was calculated and selected randomly among the staff at each center. A two-part, researcher-made questionnaire was used. The first part included questions on demographic characteristics (i.e. age, gender, marital status, work experience, job, the highest qualification, working unit and the employment status). The second part of the questionnaire consisted of 16 items including: the knowledge related to sharps injuries, complications and actions needed to be taken after an injury occurred (4 items), the history of exposure to a sharp injury and its causes if occurred (7 items), and the actions they have taken after a sharp injury occurred (5 items). The participants were asked to fill-out the questionnaire and put it in a special box which was placed for this purpose at each center. The data was collected 24 hours later. To evaluate the reliability, the test-retest method was used. The correlation coefficient was 0.93.

### 3.1. Ethical Considerations

All participants in the study signed a written informed consent to participate and were assured about the confidentiality of their personal information. Ethical issues of the study were approved in the research ethics committee in the Faculty of Nursing and Midwifery, Kashan University of Medical Sciences. Also permission was obtained from the university authorities and hospitals.

### 3.2. Data Analysis

For data analysis, descriptive and analytical statistics (chi-square, ANOVA and Pearson’s correlation coefficient) SPSS 16.0 software was used.

## 4. Results

From a total of 360 questionnaires, 298 were fully answered and were used in analysis; 68.1% (203 subjects) were female. The age ranged from 20 to 54 years, and their mean age and mean working experience were 32.25 ± 7.06 and 8.10 ± 6.63 years, respectively. No significant differences were observed between the mean age and mean working experience of men and women. Also 38.3% of the subjects (n = 114) had a history of injury from needles and sharps in the last six months. In addition, 19.8% (n = 59) subjects were injured from sharp instruments for at-least 2 times during the last six months. The mean of sharp instrument injuries was 2.74±1.56 times in the last 6 months. Most needle sticks and sharp instrument injuries have occurred among the operative nurses and midwifes and 44.8% of this group had experienced such injuries in the past six months before the study (Table 1).

**Table 1.** Demographic Characteristics of the Participants and History of Needle Sticks and Sharps Injury in the Last Six Months.

Variable	Total, No. (%)	History of Injury, No. (%)	P value
**Gender**			0.46
Female	203 (68.1)	81 (39.9)	
Male	95 (31.9)	33 (34.7)	
**Marital status**			0.80
Single	63 (22.1)	24 (38.1)	
Married	235 (78.9)	90 (38.3)	
**Job**			0.77
Nurse	155 (52)	60 (38.7)	
Lab staff	34 (11.4)	15 (44.1)	
Operating room nurse and midwife	29 (9.7)	13 (44.8)	
Nursing assistant	80 (26.8)	26 (32.5)	
**Education level**			0.20
Diploma & lower	89 (29.9)	26 (29.2)	
Associate degree	14 (6)	9 (50)	
Bachelor or higher	191 (64.1)	79 (41.4)	
**Employment status**			0.35
Permanent	66 (22.1)	29 (43.9)	
By contract	232 (77.9)	85 (36.6)	

No significant association was found between age and the history of needle stick; however, a significant correlation was observed between being exposed to needle stick and work experience. Those with more work experience had less frequent needle sticks in the past 6 months (P ≤ 0.05). In total, 32.5% of injuries from sharp instruments occurred in morning shifts. Also, 89.3% of reported that they have received hepatitis B vaccination. More than half of the participants reported that they have high levels of knowledge about the needle stick injury, its side effects and actions required when a sharp instrument injury occurs. However, no significant association was found between the level of knowledge and the history of needle stick injury (Table 2). Also, 78.7% of nurses evaluated their knowledge as high in this regard.

**Table 2.** The History of Sharp Injury in the Last Six Month and Knowledge about Needle Stick Injuries

History of Sharps Injury in the Last Six Month	Knowledge About Needle Stick Injuries, No. (%)			P value
	High Knowledge	Low Knowledge	No Knowledge	
**Yes**	75 (65.8)	30 (26.3)	9 (7.9)	0.80
**No **	111 (71.4)	35 (22.7)	9 (5.8)	

Needles were responsible for 46.5% of injuries (Table 3). Also blood sampling from a restless patient and needle recapping were the most common situations in which injuries occurred (Table 3). Also, careless interventions (in 28.25% of cases) and crowded environment in the unit (in 20.34% of cases) were cited as the two common factors predisposing to injuries (Table 3).

**Table 3.** Frequency, Type of Instrument, Behavior Leading to Injury and Predisposing Factors.

Variable	No. (%)
**Type of instrument**	
Needle	53 (46.5)
Angiocatheter	11 (9.6)
Lancet	7 (6.1)
Surgical blade	5 (4.4)
Suture needle	4 (3.5)
Vials of drugs	3 (2.6)
Others	31 (27.2)
**Behavior leading to injury**	
Injection and taking a blood sample	22 (22.2)
Needle recapping	18 (18.2)
Inappropriate disposal of needles	11 (11.1)
Preparing drugs	7 (7.1)
Transporting sharps	7 (7.1)
Others	34 (34.3)
**Predisposing factor for injury**	
Imprudence of the subject	25 (28.25)
Crowded ward	18 (20.34)
Imprudence of colleagues	10 (11.30)
Lack of facilities	5 (5.65)
Fatigue	2 (2.26)
Inappropriate education	2 (2.26)
Drowsiness	2 (2.26)
Putting needles in waste basket	5 (5.65)

**Figure 1.**
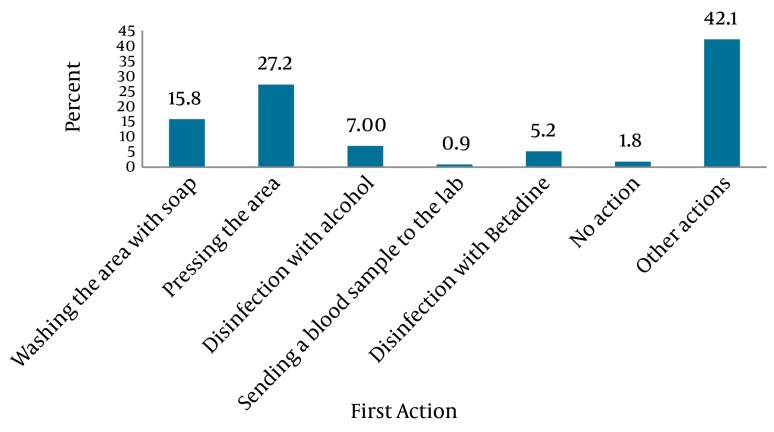
The First Action After the Injury

**Figure 2.**
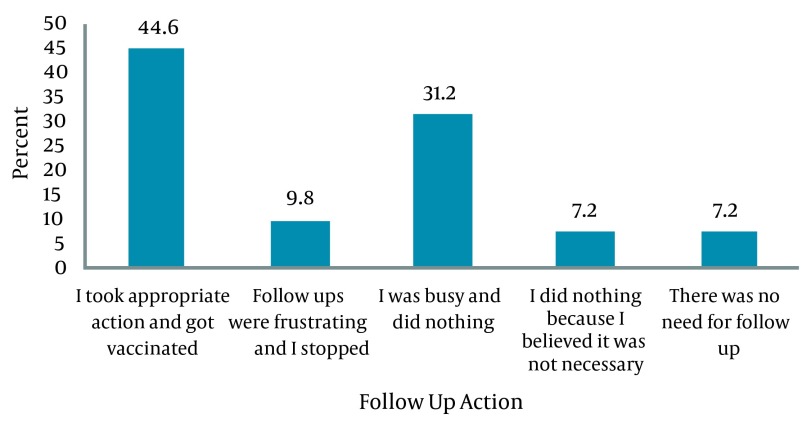
Follow Up Action After the Injury

The most common actions taken after needle stick injuries were compression (27.2%) and washing the area with soap (15.8%) respectively (Figure 1). Only 44.6% of the injured people took follow up actions after a needle stick or sharp instrument injury while no specific action was performed in 53.4% of cases (Figure 2). A significant association was found between the staff’s knowledge about sharp instrument injuries and the follow-up actions implemented after the occurrence of an injury (P = 0.01) (Table 4).

**Table 4.** Frequency of Follow-Up Measures in Different Levels of Knowledge About Needle Stick Injuries

Type of Follow-Up Action After the Injury	Knowledge About Needle Stick Injuries, No. (%)			P value
	High	Low	No	
**Yes**	40 (58.0)	7 (25.9)	3 (37.5)	0.01
**No **	29 (42.0)	20 (74.1)	6 (62.5)	

Overall, 9.6% of the staff who experienced a sharp instrument injury did not report the occurrence of the injury to the ward’s authorities; 38.6% reported the injury to the ward’s authorities and received some guidance; 24.6% of the wards authorities implemented special supportive and follow up actions. However, according to the participants, in 21.1% of cases the wards authorities were indifferent towards the staff’s injuries. Most participants (62.8%) stated that experiencing a needle stick or sharp instrument injury made them more cautious and careful.

## 5. Discussion

More than one third of the participants in this study had experienced injury to sharp instruments in the last six months. This rate was lower than that reported in other studies in Iran. The incidence of needle stick ranged from 47.05% to more than 76% in different studies previously conducted in Iran (33, 34). This rate ranged from 61% to 80% in studies conducted in Britain, Uganda and India (35, 36). Although the rate of sharp injuries in this study may be influenced by the response rate; however, the lower rate of injuries may also be attributed to the low rate of reporting (16, 17) and this may be an alarm for the need to establish a reliable reporting system for these injuries. Although the participants evaluated their level of knowledge about such injuries to be high; however, this self-rated evaluation may be inaccurate and their level of knowledge needs to be evaluated through more appropriate methods. Most operating room staff, midwives and lab staff had a history of needle stick or sharps injury in the last six months before the study. A previous study reported that such injuries are more prevalent among nurses (29). It seems that operating room staff, midwives and lab staff are at a higher risk. Hospital authorities should establish special education programs to decrease the risk in these high risk groups. Most of the participants in the present study had been vaccinated against HBV. This finding was consistent with previous studies in Hamadan Iran (37) and in Pakistan (38). Although the immunization rates in our study were acceptable, vaccination alone does not guarantee immunity against diseases. Therefore, it is necessary for high risk staff to be checked periodically for the levels of antibody of dangerous pathogens (28). In the present study, most sharp instrument injuries occurred in the morning shifts. However, no significant difference was found between working shift and occurrence of sharp instrument injury. This finding was consistent with the results of Ghasemi, who studied the frequency of needle stick injuries among healthcare workers of Ardebil hospitals (33). Also the same results were reported by Aghadoost et al., in Kashan (39), and some of the studies from other countries (40, 41). Nevertheless, Ayas et al., studied the risk of self-reported percutaneous injuries in interns and reported that most injuries occurred during the night shifts (42). In the current study, injecting and taking a blood sample from a restless patient and recapping the needles were the most dangerous interventions resulting in needle stick injury. This finding was consistent with several previous studies (17, 29, 37), but are inconsistent with Jayanth et al., (17). Also as reported in several studies (29, 42) the most common damaging instrument in the current study were needles and intravenous catheters. However using safe syringes, needles and intravenous catheters can significantly reduce the rate of needle stick injuries. Modern safety syringes, needles and intravenous catheters may decrease injury. In the present study, the most common causes of injury were imprudence and crowded ward environment. Several previous studies also reported that imprudence of the healthcare staff, high workload, and rushing were common predisposing factors for needle stick injuries among nurses and other healthcare workers. Also disposal of needles with other rubbish is an important risk factor for needle stick injury among the custodian and ward cleaners (29, 42-44). In the present study, the most common actions performed after a needle stick injury were applying pressure and washing the area with soap and water. These findings are consistent with the results of Rakhshani et al., and Baghcheghi et al. (28, 29). But, Hashemi et al., reported that most needle stick injured staff immediately referred to the hospital’s center for infection control to receive proper treatment (37). It seems that executing proper in-service education programs along with establishing protocols for implementing them following needle stick injuries may help healthcare workers receive proper treatment.

More than a half of the healthcare workers with needle stick or sharps injury refused follow-up for various reasons. Also, facilities for treatment and follow up was available in less than a quarter of the cases, The exact rate of injury may be underestimated.
